# PB.10. Stereotactic 20 mm basket Intact Breast Lesion Excision System biopsy for indeterminate breast microcalcification: pilot study within a UK breast unit

**DOI:** 10.1186/bcr3719

**Published:** 2014-11-03

**Authors:** A Leaver, AJ Potterton, S Athey, CM Lee, S Sharma, A Redman, D Hemming, L Lunt

**Affiliations:** 1 Queen Elizabeth Hospital NHS Trust, Gateshead, UK

## Introduction

For several years our breast unit has evaluated indeterminate breast microcalcification using stereo-guided vacuum-assisted core biopsy (VACB) - piecemeal acquisition of unorientated tissue. The Intact Breast Lesion Excision System (Intact) removes a single larger piece of tissue, potentially allowing pathologists to calculate margins and visualise lesion architecture more easily. This study evaluates the role of the 20 mm Intact in assessment of subcentimetre clusters of indeterminate breast microcalcification.

## Methods

The radiology authors performed the Intact biopsy on subcentimetre clusters of indeterminate, sonographically invisible breast microcalcification. A prospective audit was performed, with pathology information from the multidisciplinary meeting. Technical considerations encountered during use of the Intact, sample pathology and short-term patient outcomes are described.

## Results

The Intact was performed on 23 female patients, was technically straightforward to operate and was well tolerated by patients. Metal fragments from basket removal were radiologically confused with calcification, but did not result in patient mismanagement.

Pathologists found specimens easier to interpret than VACB samples, although one (1/23) specimen had a 2.5 mm area of diathermy artefact that could potentially have obscured a lesion. Three (3/23) cases of ductal carcinoma *in situ *(DCIS) were identified, post-biopsy radiographs consistent with complete excision of microcalcification. The Intact specimen pathology review showed incomplete excision of disease and two of the three cases of DCIS had further DCIS on surgical wide local excision. No invasive disease was identified in any study patient.

## Conclusion

In this small pilot study, the Intact has been a reliable and effective tool for stereotactic diagnostic biopsy of breast microcalcification.

